# A strategy for entomo-virological surveillance of yellow fever, dengue, zika, and chikungunya viruses in field-collected mosquitoes

**DOI:** 10.1016/j.mex.2023.102356

**Published:** 2023-09-01

**Authors:** María-Angélica Calderón-Peláez, Juan S. Mantilla-Granados, Myriam Lucía Velandia-Romero, Eliana Calvo, Jaime E. Castellanos, Víctor Alberto Olano

**Affiliations:** a Vicerrectoría de Investigaciones, Grupo de Virología, Universidad El Bosque, Bogotá 110121, Colombia; b Vicerrectoría de Investigaciones, Saneamiento Ecológico, Salud y Medio Ambiente, Universidad El Bosque, Bogotá 110121, Colombia

**Keywords:** Mosquito collection, Arbovirus detection, Multiplex PCR, Entomo-virological surveillance of emerging arboviruses

## Abstract

Arboviruses transmitted by Culicidae insects are significant threats to human health, presenting dynamic transmission cycles and involving different vectors and hosts. The surveillance and characterization of the vectors involved in these cycles are crucial for understanding and preventing potential outbreaks. Therefore, we propose a strategy that we used for entomological surveillance of urban, rural, and sylvatic mosquitoes and to characterize natural infection by four major arboviruses.

• Immature and adult mosquitoes were collected intra, peri and extradomicilie of urban and rural households, using different collection methodologies.
• Mosquitoes were pooled or separated in head-thorax and abdomen, according to the species.
• A multiplex nested RT-PCR (Reverse transcription polymerase chain reaction) method was used for the simultaneous detection of dengue virus (DENV), zika virus (ZIKV), chikungunya virus (CHIKV), and yellow fever virus (YFV).

Overall, this strategy proved helpful for vectors surveillance at different ecosystems, as well as for implementing a low-cost molecular surveillance system that allows the early detection of potential outbreaks, and identify other potential vectors involved in viral transmission.

## Graphical abstract

**Figure:**
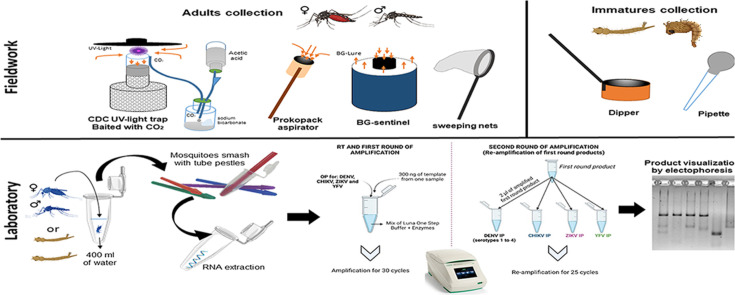

Specifications table

**Table:** 

Subject area:	Biochemistry, Genetics and Molecular Biology
More specific subject area:	Virology, Medical Entomology
Name of your method:	Entomo-virological surveillance of emerging arboviruses
Name and reference of original method:	Calvo E, Sánchez-Quete F, Durán S, Sandoval I, Castellanos J. Easy and inexpensive molecular detection of dengue, chikungunya and zika viruses in febrile patients. Acta Trop. 2016; 163: 32–7. doi:10.1016/j.actatropica.2016.07.021.
Resource availability:	N.A.

**Method details**

## Sample size

The sample size was calculated using the software OpenEpi version 3 and the open code calculator SSPropor, with values of 90% confidence, and 5% error, using the projected 2005 population data for the urban area [1], the 2014 census for the rural areas [2] and the reported dengue prevalence in the urban area (Secretaría de Salud del Meta, personal communication). A total of 178 urban households were selected, covering nine neighborhoods, and 97 rural living places, distributed in 8 rural settlements (Agua Azul, Altamira, El Billar, La Florida, La Esperanza, Las Delicias, Los Andes, and San José). Details of the geographical distribution of the sampling points could be consulted at Mantilla-Granados et al., 2022. Such settlements were selected based on their predicted high risk of yellow fever transmission (Secretaría de Salud del Meta, personal communication; [3]), the feasibility of ground transportation, and the low risk of public order issues. For the urban area, blocks were numbered, and 22 were randomly selected using Google Earth, and Excel and the residents were invited to participate in all households. For the 8 rural settlements, we attempted to visit all the households, after socializing the project with local leaders or holding general meetings with the community.

## Mosquito and immature forms collection

Samples were collected by visiting each household during the day (8:00–18:00) from August to November 2019. Each visit took an average of 30 min. Upon signature of the informed consent form at each house, a signed copy was given to the participants, and the team collected mosquitoes accompanied by at least one inhabitant. Each household was visited only once, except for those selected for the placement of an entomological trap, these sites were visited at least twice, just to check or remove the traps.

Immature mosquito collections were carried out in all artificial and natural water-holding containers found in three categorized areas: intradomicile (inside the house), peridomicile (0–10 m away from the household (backyard, front yard, and garden), and extra-domicile (10–500 m away from the household).

While one researcher inspected the house for the presence of breeding sites, and attempted to collect all pupae and larvae, when the larvae density was too high (more than 100 individuals), at least ten percent of larvae from these containers were collected, using a dipper or a pipette, depending on the size of the potential breeding site. Larvae and pupae were stored in 50 mL tubes with the container's water to keep them alive until pupae emerged to detect newborn infected females or males. Another researcher collected adult resting mosquitoes using a Prokopack aspirator [4] in the intradomicile and peridomicile for 15 min each.

Extradomiciliary sampling was carried out along longitudinal transects of 0.5 to 1 km, using two simultaneous sweep nets (to catch mosquitoes resting on vegetation or to catch active mosquitoes). We initially tried to use Prokopack aspirators, but they didn´t work well in open areas, driving away the mosquitoes. The transects were carried out in different areas around rural houses within forest patches, especially in forest glades, which we entered with the agreement of the landowners.

At the same time, two other researchers were looking at the nearby position of the transects (two to five meters) for the presence of potential breeding places, and immature mosquitoes were collected with 3 ml or 5 ml plastic pipettes, depending on the size of the breeding sites. The collected material was kept alive as the one of intra- and peridomicile. As the size of the forest paths varied from 10000m^2^ to 100000m^2^, we tended to sample as much of the accessible area as possible, sampling approximately for one to two hours, depending on the forest patch size and the number of collectors, standardizing the sampling effort as collected material per person and sampling time.

**Fig. 1:**
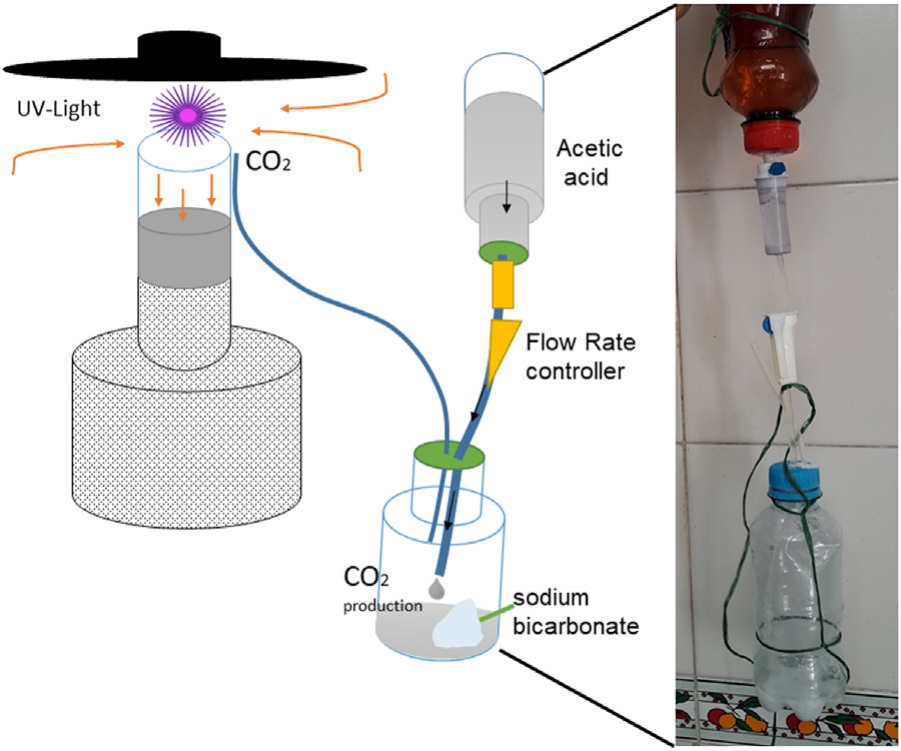
CDC trap with UV light and the device for CO2 production. (A). Schematic representation. (B) Photography of the device.

Adult mosquitoes were stored, labeled, and transported in plastic containers inside a polystyrene cooler box with blue ice packs. Occasionally, when the households were close to rivers or forest areas, and inhabitants allowed, we also used I), two CDC miniature light traps model 2506 N (Curtis Dyna-Fog's Ltda, USA) baited with CO_2_ (using a partially controlled chemical reaction between 5% acetic acid and sodium bicarbonate in a special container consisting of two pair bottles connected to a micro drop Infusion Set with Flow Rate Controller, Fig. 1), and II) four BG-sentinel baited with a BG-Lure (Biogents AG, Germany) traps to catch adult mosquitoes in the intra- and peri‑domicile. One trap of each type was deployed in each selected household for 48 h in the case of the BG- sentinel traps and 24 h for the CDC traps (nights only). To avoid potential interference, the traps were spaced at least 100 m apart and one to two meters above the ground; in the case of baited traps with CO_2_ traps, a distance of at least 140 m is recommended [5]. In addition, we visited each selected household every 12 h to change the collection bag and replace the trap batteries, each house was visited once, and sampling was conducted daily. A total of 22 CDC and 37 BG bags were collected, with each bag considered as an independent collection unit.

## Mosquito identifications

Once in the field lab, the adults material was stored at −20 °C until taxonomic identification, the alive immature material was checked twice at day to remove dead larvae or pupae which were identified immediately or stored in 70% ethanol; reared adults were kept dry, labelled and preserved as other adult material. The taxonomic identification was done in field using stereomicroscope Zeiss™ Stemi™ DV4 (Jena, Germany), with different taxonomic keys [[6], [7], [8]] to identify most of the individuals to species level. Some representative mosquitoes of each species were mounted and deposited in the Museum of Sciences of Universidad El Bosque (accession numbers MCUB-R-HE-DI-000,518 to MCUB-R-HE-DI-000,561). Once identified, two to thirty *Ae. aegypti* individuals were pooled by developmental stage, sex, date, and location and preserved in RNAlaterⓇ (Thermo Fisher Scientific, Waltham, Massachusetts U.S.A). The females of *Haemagogus janthinomys* and *Aedes serratus* were segmented in abdominal and head-thorax regions using micro-scissors (heat sterilized in a flame), and preserved individually in RNAlaterⓇ. Males and immature mosquitoes were processed as *Ae. aegypti* samples. Finally, the material collected was transported to Bogotá to the Virology Group laboratory of the Universidad El Bosque and stored at −80 °C.

## RNA extraction

Samples were thawed on ice, and then the individuals were transferred to tubes containing 400 µl diethyl pyrocarbonate treated water (Sigma-Aldrich, D5758), using a curved-tip syringe needle . The specimens were carefully crushed with microcentrifuge tubes pestles (USA Scientific, 1415–5390). The supernatants (without debris) were then transferred to the viral RNA extraction tubes of the RTPⓇ DNA/RNA Virus Mini kit (Stratec, 1,040,100,300) and processed according to the manufacture's protocol. Briefly, the extraction tubes were heated to 65 °C for 15 min, and then 95 °C for 10 min. The lysates were mixed with the binding solution and transferred to the minicolumn; after two washes, the DNA/RNA was eluted with the provided buffer. Samples were then quantified using the Nanodrop system (Implen) and used as templates in RT-PCR reactions or stored at −80 °C until use.

## Viral rna amplification and method validation

**Fig. 2:**
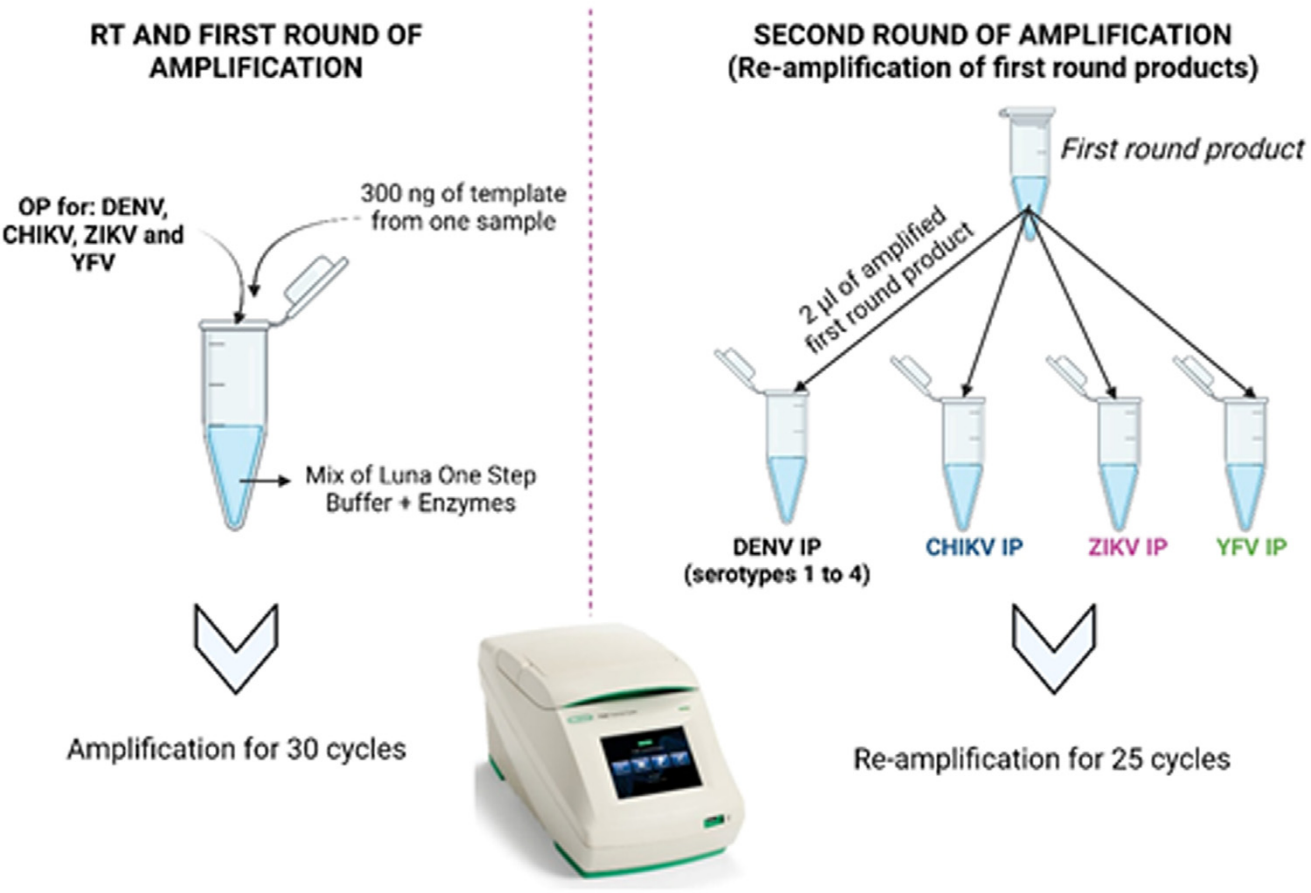
Schematic representation of multiplex semi-nested RT-PCR. For the RT and the first round of amplification, the outer primers (OP) are mixed in one reaction tube per sample and the template is amplified for 30 cycles. The first round product then becomes the template for the second round of amplification using the inner primers (IP) for each virus in separate reactions and the sample is re-amplified for a further 25 cycles.

For viral RNA detection, a semi-nested PCR amplification was performed (Fig. 2) following the protocol described by Calvo et al., [9]. Briefly, retro-transcription and amplification of viral RNA was performed using LunaⓇ Universal kit One-Step RT-PCR, following the manufacturer's instructions (Thermo Fisher Scientific), 0.2 µM primers outer (four pairs) and 100–400 ng of purified RNA as template. As a negative control, we use nucleotide-free water instead of the sample in the PCR mix. For the detection of DENV, ZIKV and CHIKV we used the primers described by Calvo et al., [9] and for YFV detection, primers were designed for this study in (Table 1).

**Table 1:** Primers used for the detection of YFV. The outer reverse primer (OR) acts as the inner reverse primer (IR) in the PCR second-round (labeled in bold).

Outer primers (O)	OF GCTAATTGAGGTGYATTGGTC **OR CGAACTCCTCGTCGTACCATA**	205 bp
Inner primers (I)	IF CAAATCGAGTTGCTAGGCAAT **IR CGAACTCCTCGTCGTACCATA**	145 bp

The amplification program included a retro-transcription (RT) step at 50 °C for 15 minutes (min), an initial denaturalization at 95 °C for 2 min, and then 30 cycles of 95 °C for 30 seconds (s), annealing at 55 °C for 30 s and extension at 72 °C for 30 s, then a final extension at 72 °C during 5 min.

Importantly, in none of the cases was it possible to detect the product on the electrophoresis gels, demonstrating the need for a second-round of PCR. For this, each virus was amplified in a separate reaction. DENV PCR reaction included five internal primers (one forward and four reverse, one for each DENV serotype), while CHIKV, ZIKV, and YFV reactions contained one inner primer pair. The later means that four different PCR reactions were performed for each sample, using 2 µl of the first-round amplicon as a template. The amplification protocol comprised an initial denaturation at 95 °C for 5 min and then 25 cycles of 95 °C for 30 s, 60 °C for 30 s, and 72 °C for 30 s, followed by a final extension at 72 °C for 5 min.

**Fig. 3:**
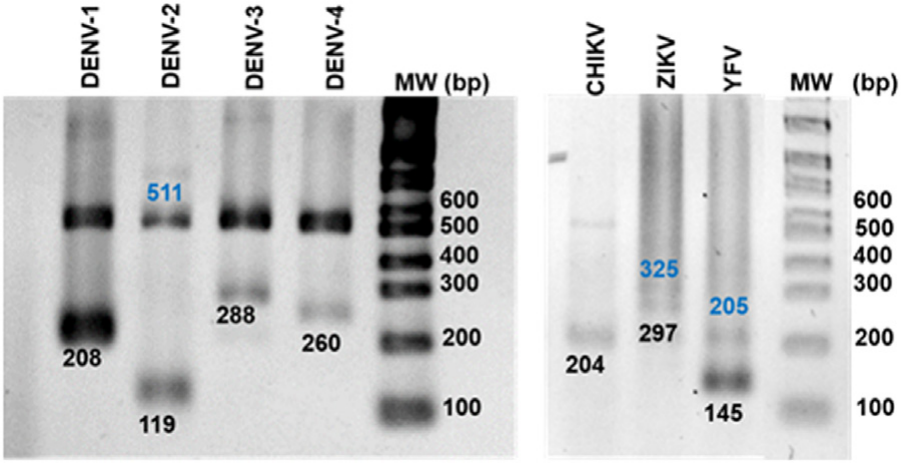
Arbovirus detection in RNA extracted from culture-harvested control viruses. Second round product analysis by electrophoresis on an agarose gel. First round products sizes are shown in blue, and second round products are shown in black.

The second-round products were then separated on 2% agarose gels, at 90 V for 45 min and then stained with ethidium bromide (Sigma-Aldrich) for 10–20 min. Finally, gels were visualized using the BioRad Gel Doc XR Plus Imaging System. For positive controls or viral RNA extracted from supernatants of c6/36 cells infected with each virus, two bands corresponding to the first- and second-round products were detected (Fig. 3). Negative controls for each round of amplification, which would indicate some form of cross-contamination, as well as positive controls were used in each run of samples collected in the field.

**Fig. 4:**
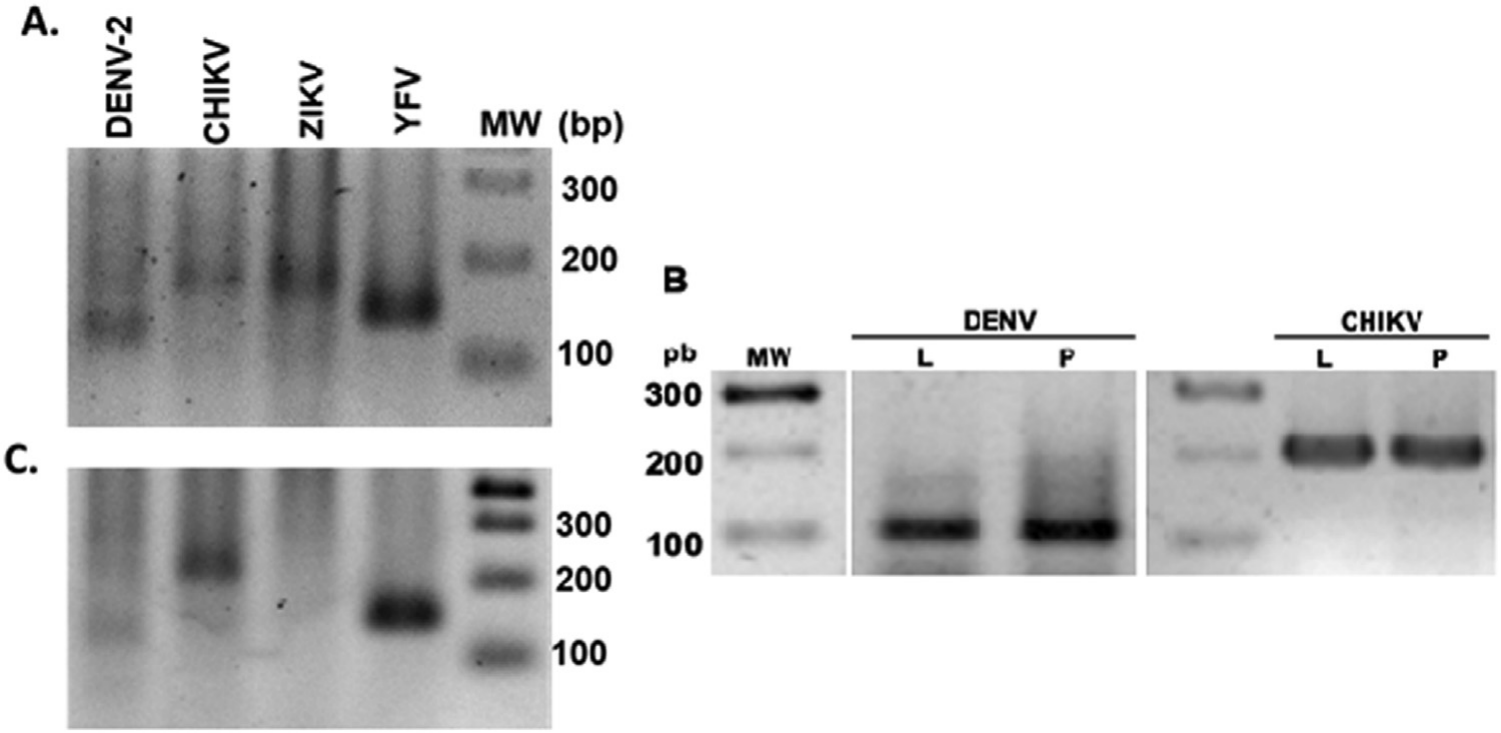
Arbovirus detection in RNA extracted from field-caught mosquitoes and immature forms of *A. aegypti*. (A) Using 300 ng of RNA, we detected positive samples for DENV serotype 2, CHIKV, ZIKV, and YFV in mosquito pools. (B). Immature forms larvae (L) or pupae (P) positive for DENV2 and CHIKV. (C). Abdomens of segmented females positive for DENV2 and CHIKV.

This strategy allowed us to detect DENV, ZIKV, CHIKV, and YFV infections (single or multiple) in individual whole mosquitoes (adults, larvae, or pupae), using a minimum amount of RNA (100 ng) (Fig. 4). However, to ensure the detection of the virus in all of the samples and to avoid possible false negatives, we used 300 ng of RNA in the subsequent experiments.

In the case of segmented females (head/thorax and abdomen), the amount of RNA obtained ranged from 50 to 80 ng/µl, for these samples, it was always necessary to use 300 ng of template for the first round of amplification, to increase sensitivity and achieve detection; some of these samples were positive for one or more viruses (Fig. 4C).

To confirm the presence of YFV, amplicons obtained from positive samples were sequenced by Sanger method, and the sequences were analyzed by BLASTⓇ (NCBI, https://blast.ncbi.nlm.nih.gov/), against previously reported sequences.

## Ethics statements

Prior to data collection, a written informed consent form, regarding the entomological collections, was explained to the local residents as approved by the Ethic´s Committee of Universidad El Bosque, Bogotá, Colombia (Acta 006–2019. March 14 of 2019). Sampling was authorized by National environmental agency (ANLA) (Resolution 0198, 2016; modified with resolution number 01,470, of November 17, 2017).

## CRediT authorship contribution statement

**María-Angélica Calderón-Peláez:** Conceptualization, Methodology, Validation, Formal analysis, Investigation, Writing – original draft, Visualization, Writing – review & editing. **Juan S. Mantilla-Granados:** Conceptualization, Methodology, Validation, Formal analysis, Investigation, Writing – original draft, Visualization, Writing – review & editing. **Myriam Lucía Velandia-Romero:** Conceptualization, Supervision, Resources, Writing – review & editing, Funding acquisition. **Eliana Calvo:** Writing – review & editing. **Jaime E. Castellanos:** Supervision, Writing – review & editing. **Víctor Alberto Olano:** Supervision, Resources, Writing – review & editing, Project administration, Funding acquisition.

## Declaration of Competing Interest

The authors declare that they have no known competing financial interests or personal relationships that could have appeared to influence the work reported in this paper.

## Data Availability

Data will be made available on request.
